# 513 Fluid Over-Resuscitation in Burn Patients After Initial 24-Hours of Care is Associated with Increased Mortality

**DOI:** 10.1093/jbcr/irac012.144

**Published:** 2022-03-23

**Authors:** Sami K Kishawi, Avanti Badrinathan, Casey L Kohler, Justin E Dvorak

**Affiliations:** MetroHealth Medical Center, Cleveland, Ohio; University Hospitals Cleveland Medical Center, Cleveland, Ohio; MetroHealth Medical Center, Cleveland, Ohio; MetroHealth Medical Center, 44120, Ohio

## Abstract

**Introduction:**

Fluid resuscitation is a cornerstone of modern burn care. Despite the use of well-established formulae to determine the appropriate amount of fluid resuscitation for the first 24 hours of care, there is increasing recognition that patients receive fluids in excess of predicted volumes, a phenomenon termed fluid creep. Underscoring the significance of this phenomenon is the association between large volumes of fluid resuscitation and adverse outcomes. Although research in non-burn ICU patients reveals a clear association between overall fluid intake and increased morbidity, minimal burn-related literature exists regarding fluid patterns after the initial 24-hour period and their impact on outcomes. We hypothesized that increased fluid administration after the standard initial resuscitation period is associated with increased morbidity and mortality.

**Methods:**

A retrospective chart review was performed for 113 patients with ≥20% TBSA burns admitted to an American Burn Association-verified burn center between 2010 and 2020. Patients admitted with Stevens-Johnson Syndrome and/or Toxic Epidermal Necrolysis, with length of stay ≤ 72 hours, who required renal replacement therapy (RRT) within 72 hours of admission, and those with withdrawal of care ≤ 7 days of admission were excluded. Univariate and multivariate logistic regression was used to determine the association between the primary outcome of in-hospital mortality and secondary outcomes of increased ventilator days, acute kidney injury, need for RRT, and hospital length of stay, with increasing total and net fluid volumes from days 2 through 7 of treatment. Additionally, the association between first OR day and total fluid volumes in the first week were assessed.

**Results:**

Median age was 41 years (IQR 23-55) and TBSA was 31% (IQR 24-43). 21 patients (18.6%) died during hospitalization. Increase in net fluid balance from days 2-7 were associated with increased mortality (OR 1.016, 95% CI 1.00 – 1.03, *p* = 0.013). Increasing total fluid volumes were significantly associated with increased ventilator days (OR 1.027, 95% CI 1.008-1.047, *p* = 0.006) and acute kidney injury (OR 1.003, 95% CI 1.000-1.006, *p* = 0.017). Early first OR day was associated with decreased net fluid balance between hospital days 2-7 (OR 0.993, 95% CI 0.989-0.997, *p* = 0.001).

**Conclusions:**

Similar to studies on other ICU populations, increasing total fluid volumes and net fluid balance is associated with adverse outcomes in critically ill burn patients. Additionally, earlier initial OR is associated with less total fluid volumes and lower net fluid balance in the first week of hospitalization. Further investigation is needed to elucidate optimal markers of resuscitation in burn patients in an effort to decrease adverse fluid administration.

